# Global research in schizophrenia and serotonin: a bibliometric analysis

**DOI:** 10.3389/fpsyt.2024.1436906

**Published:** 2024-08-02

**Authors:** Gustavo Canul-Medina, Gael López-Pech, Francisco Jiménez-Trejo

**Affiliations:** ^1^ School of Medicine, Educational Center Rodriguez Tamayo, Ticul, Yucatan, Mexico; ^2^ Cellular and Tissue Morphology Laboratory, National Institute of Pediatrics, Mexico City, Mexico

**Keywords:** schizophrenia, serotonin, bibliometric analysis, scopus, VOSviewer

## Abstract

**Background:**

Schizophrenia is a chronic mental illness that affects millions of individuals worldwide. The etiological origin of schizophrenia is heterogeneous, but it has been shown to be associated with dysfunction in serotonin activity, serotonin receptors, and serotonin metabolism in the brain. Bibliometric analysis is a tool used to scrutinise and analyse research activities and evidence in a specific research area. No existing bibliometric analyses have considered both serotonin and schizophrenia.

**Methods:**

We conducted a bibliometric analysis including 12,027 studies related to the schizophrenia–serotonin link published from the inception of the study to 2023 and available in the Scopus database. We used VOSviewer software to identify global trends, analyse the author and editors keywords, the most cited articles and author, as well as the most productive institutes and journals publishing research on schizophrenia–serotonin link.

**Results:**

Most publications related to the link between schizophrenia and serotonin are focused on adult humans and examine topics such as antipsychotic agents, depression, and serotonin uptake inhibitors. The *Journal of Clinical Psychiatry* has published the most papers on the schizophrenia–serotonin relationship. Among nations, the United States is the leader in publications. King’s College London is the institution with the highest number of publications, and H. Y. Meltzer is the most influential author. Growing trends in schizophrenia–serotonin research are personalised medicine, alternative medicine, transcranial magnetic stimulation, artificial intelligence, nervous system inflammation, brain-gut axis, and the gut microbiome.

**Conclusion:**

Since 1950, there have been several fluctuations in the number of published studies related to schizophrenia and serotonin. We believe that the development of novel medications and treatments for schizophrenia will be increased in the future, as well as research into genetic risks, psychological factors, and cranial neuroimaging components. Future schizophrenia and serotonin research is likely to focus on personalised medicine, alternative therapies, novel pathogenesis of schizophrenia, and the use of emerging technologies such as artificial intelligence.

## 1 Introduction

**Figure 1:**
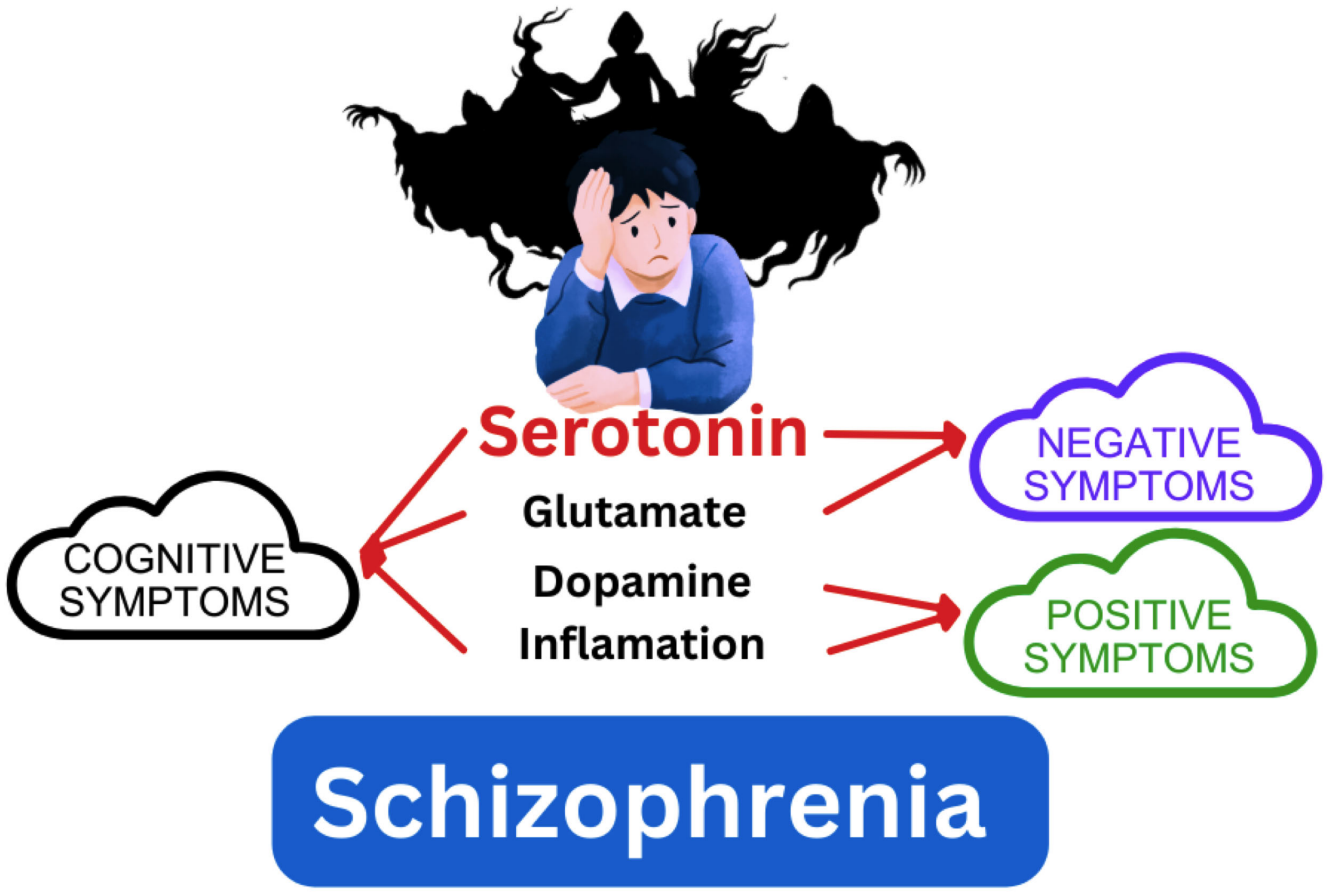
Relationship between serotonin and other neurotransmitters in schizophrenia.

Schizophrenia is a chronic mental illness of the central nervous system that affects millions of people worldwide and triggers psychosis and disability. The global prevalence of schizophrenia is around 1% (1). Between 1990 and 2019, the number of people with schizophrenia rose from 14.2 to 23.6 million (2). The etiological origin of schizophrenia is heterogeneous. However, the literature has documented that dysfunction in serotonin and other neurotransmitters might participate in schizophrenia development (**Figure 1**) (3).

Serotonin (5-hydroxytryptamine;5-HT; C_10_H_12_N_2_O) is a molecule with central and peripherical functions in the human body such as regulating mood and cognitive functions (4). Imbalances in serotonin concentration have been linked to a range of health conditions such as depression and schizophrenia (5). The main relationship between serotonin and schizophrenia is that changes in serotonin concentration can contribute to the onset of schizophrenia, and conversely, schizophrenia can gradually affect the serotonergic systems of the brain (6). Nowadays, there is no consensus on which one comes first. Serotonin is a molecule synthesized predominantly in peripheral tissues by tryptophan hydroxylase-1 (TPH1) and in the central nervous system by tryptophan hydroxylase-2 (TPH2), both from amino acid L-tryptophan (L-Trp).

Diverse studies have shown an association between increased concentrations of the serotonin metabolite 5-hydroxyindoleacetic acid (5-HIAA) in cerebrospinal fluid (CSF) and family history of schizophrenia (7). In addition, evidence has demonstrated that the 5-HT_1A_, 5-HT_2A_, 5-HT_2B_, 5-HT_6_, 5-HT_7_ serotonin receptors are modified in patients with schizophrenia (8–12). Therefore, serotonin is a medical target for many psychiatric and neurological disorders, including antipsychotic treatment (antipsychotic atypical, typical, SSRI, MAOI). Notwithstanding the increasing evidence that dysfunction of serotonin activity in the brain is strongly associated with schizophrenia, other neurotransmitters such as dopamine and glutamate, or other metabolites in the L-Trp metabolism, as well as kynurenine pathway and its rate-limiting enzymes, might also participate in generating schizophrenia (13–15).

Bibliometric analysis (BA), which primarily focuses on academic productivity, is a tool used to scrutinize and analyse research activities and evidence in a specific research area. These methods allow scientists to analyse the fragmented literature that is available in specific research areas, create a comprehensive overview, examine collaborations, identify emerging trends in journals, evaluate article performance, recognise research gaps, and generate new research ideas (16–18). It considers hundreds or even thousands of published scientific works (research articles, books, conference proceedings, etc.) from databases like Scopus, NCBI and Web of Science. Furthermore, BA analyses journal details, author collaborations, sponsors, university affiliation, and the occurrence of keywords within articles. Nonetheless, BA does not achieve synthetic knowledge of a specific research area like other methods such as meta-analyses or systematic reviews (19, 20).

Several bibliometric analyses have explored the trends in research on the association of schizophrenia with other diseases (i.e. inflammation, oxidative stress), but none has considered serotonin and schizophrenia together. This bibliometric analysis aims to explore research activities of schizophrenia in the context of serotonin to identify the current state in this field. Our work aspires to recognize emerging trends that can help shed light on potential ideas for future research on the schizophrenia-serotonin field.

## 2 Methods

The data used in this bibliometric analysis were downloaded from Scopus, which is the largest database of indexed publications (21).

### 2.1 Search strategy

**Figure 2:**
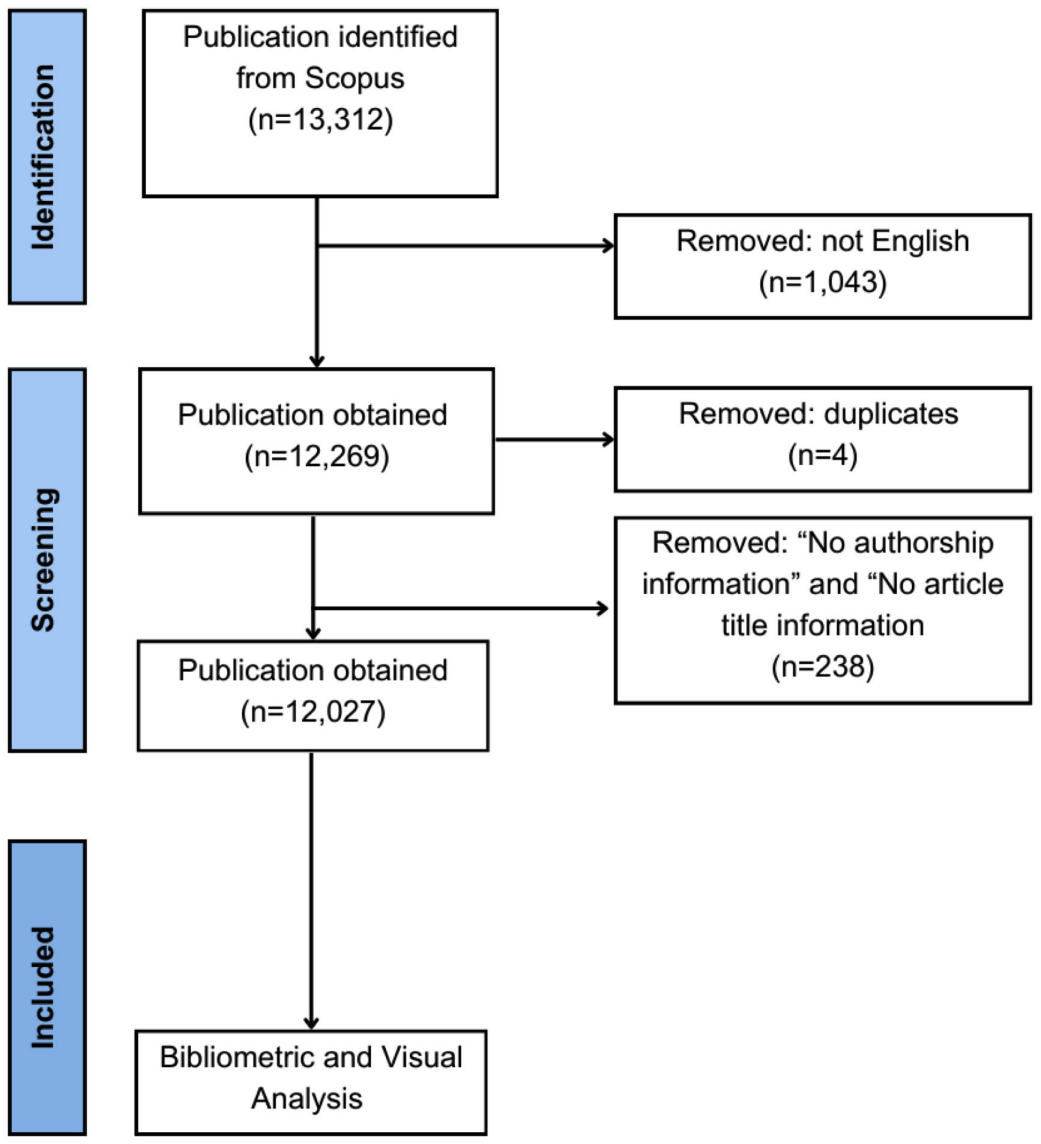
Flow diagram of literature search and selection process in the bibliometric analysis.

The **Figure 2** show the flow diagram of literature search and selection process in our bibliometric analysis.

We used the terms ‘schizophrenia’ and ‘serotonin’ and related synonyms as search terms in Scopus. To reduce the presence of false positive search results, we used *keywords search* as our search method strategy (22). Keyword search include the author keywords and journal editors keywords. The following search strategy were used: KEY (“schizophren” OR “schizophrenia” OR “schizophrenias” OR “schizophrenic” OR “schizoaffective” OR “psychosis” OR “psychotic”) AND KEY (“5ht” OR “5-ht” OR “serotonin” OR “5-hydroxytryptamine” OR “5hydroxytryptamine”). Literature published from the inception of the study to 2023 was analysed. In our use of Scopus, we defined the literature as “all types”. All documents were downloaded on 4 February 2024 in RIS and CSV formats. We used OpenRefine (Creative Commons PO Box 1866, Mountain View, CA 94042, United States) to clean and harmonize raw data downloaded from Scopus to ensure the accuracy and reliability of the results (23).

### 2.2 Data analysis

Mapping scientific knowledge is essential for bibliometric analysis and facilitates the identification of trending and emerging topics within a particular area of study while supporting strategic decision making for future research (24, 25). We used VOSviewer (Centre for Science and Technology Studies, Leiden University, Leiden, The Netherlands) to visualize the networks and create maps of the most common author keywords and journal editors keywords of the retrieved documents, the most cited articles and author, as we all the most productive institutes and journals publishing research on the schizophrenia–serotonin link. Threshold values and visualization methodologies for each type of analysis in VOSviewer are found in the **Supplementary Material** (see, **Supplementary Table 1.3**).

The Impact Index Per Article (the top 10 most highly cited papers) was obtained from Reference Citation Analysis (RCA) (Baishideng Publishing Group Inc. Pleasanton, CA 94566, United States). The H-Index Author was obtained from Scopus. The H-Index Journal was obtained from Scimago Journal and Country Rank (Scimago Research Group, S.L., Granada, Spain). GraphPad Prism 9 (GraphPad Prism Software Inc., San Diego, CA, USA) was used to make line charts.

## 3 Results

### 3.1 Types of publications

A total of 12,027 documents published were obtained from the Scopus database and analysed. Nine different types of publications were represented in the sample. Research articles comprised 49.38% of our sample (n = 5,939), and reviews made up 34.30% (n = 4,125). **Table 1** lists the different document types and the proportion of the sample made up by each.

**Table 1:** The categories of retrieved documents in Scopus associated with schizophrenia and serotonin.

Type of Document	Articles	Proportion (%)
Article	5,939	49.38%
Review	4,125	34.30%
Editorial	515	4.28%
Book chapter	440	3.66%
Letter	431	3.58%
Notes	238	1.98%
Short survey	185	1.54%
Conference paper	152	1.26%
Others	2	0.02%
Total	12,027	100

### 3.2 Number of publications and temporal evolution

**Figure 3:**
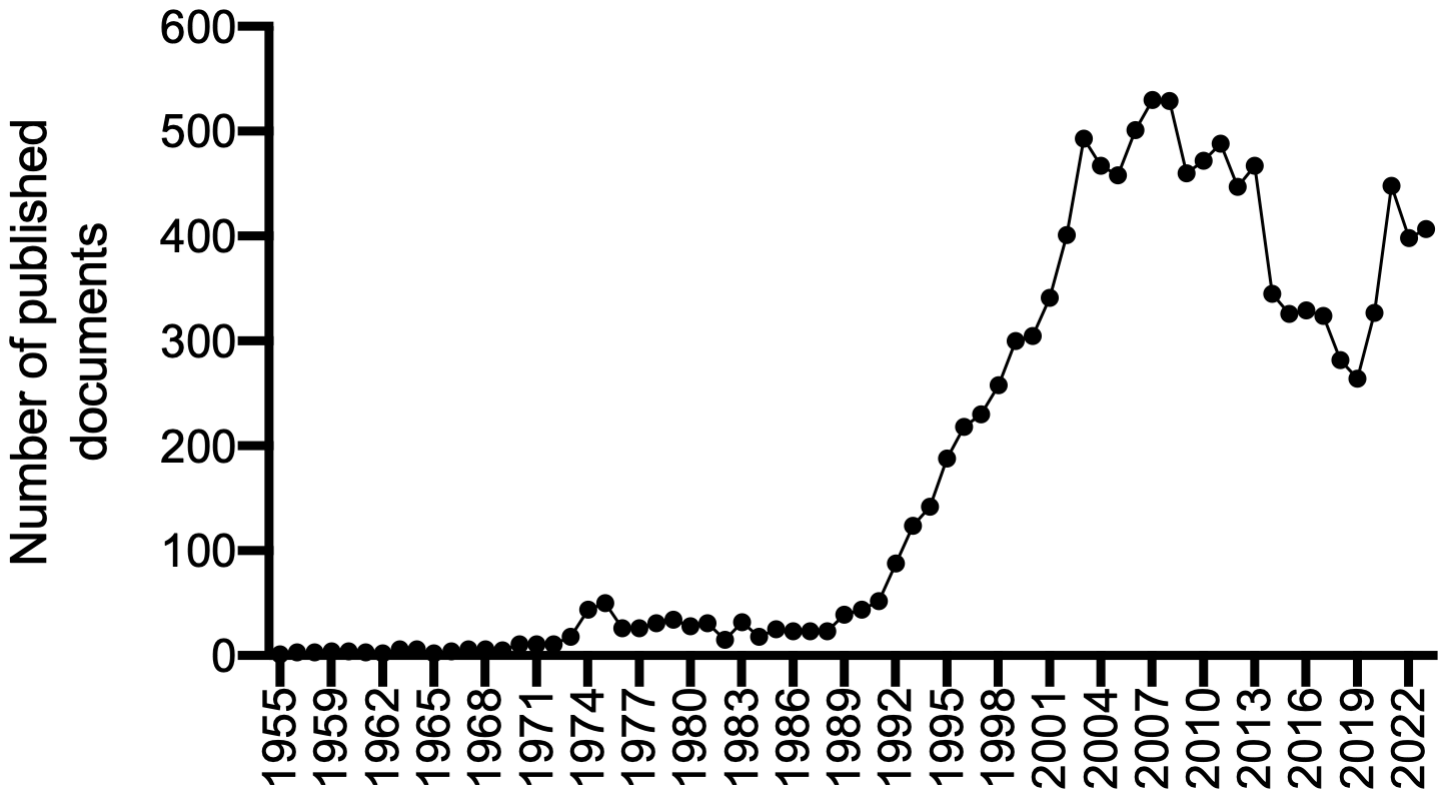
The annual trends in publications associated with schizophrenia and serotonin.

**Figure 3** shows the annual trends in the number of publications concerning the schizophrenia–serotonin link. Since the first article was published in 1955, there have been several fluctuations in the volume of research related to schizophrenia and serotonin. Research on this topic experienced limited but steady growth, increasing from one article in 1955 to five in 1969 (growth rate: 12.2%). During the following two decades, research on these topics grew from 15 articles in 1970 to 44 in 1990 (growth rate: 20.8%). The growth rate is calculated as follows: annual growth rate = ( present publication/past publication)^1/n^ – 1 ) x 100, where n = number of time periods.

In the 1990s, the amount of research related to schizophrenia and serotonin began to increase, rising from 52 articles in 1991 to its peak at 529 articles in 2008. During the 1990s, the growth rate was 24.5%; it was 7.1% between 2000 and 2008. In 2008, research related to schizophrenia–serotonin suddenly began to decrease, dropping to 265 articles in 2019 (growth rate: −6.1%). However, research related to schizophrenia–serotonin began to rise again during the global coronavirus (COVID-19) pandemic, increasing from 269 articles in 2019 to 407 in 2023. It reached a local maximum value of 448 articles in 2021 (growth rate: 10.9%). The predictive curve (R^2^ = 0.738) shows projected growth in publications related to the association between schizophrenia and serotonin from 2023 to 2033, with an expected 533 articles per year in 2033.

### 3.3 Countries and publications

**Table 2** lists the ten countries that have been the most active in schizophrenia–serotonin research. With 4,918 publications (33.65%), the United States tops the list of nations; it is followed by the United Kingdom with 1,428 publications (9.77%) and Canada (5.28%).

**Table 2:** The top ten countries and institute/university publishing research associated with schizophrenia and serotonin from 1955 to 2023.

Rank	Country	Articles	Proportion (%)
1	United States	4,918	33.65
2	United Kingdom	1,428	9.77
3	Canada	772	5.28
4	Germany	713	4.88
5	Italy	682	4.67
6	Australia	553	3.78
7	Japan	523	3.58
8	Spain	395	2.70
9	France	389	2.66
10	Netherlands	316	2.16
Rank	Institute/University	Articles	Country
1	King’s College London	304	United Kingdom
2	University of Toronto	267	Canada
3	Harvard Medical School	262	United States
4	VA Medical Center	220	United States
5	Centre for Addiction and Mental Health	177	Canada
6	University of Melbourne	169	Australia
7	University of California, San Diego	164	United States
8	Yale School of Medicine	163	United States
9	National Institute of Mental Health	158	United States
10	Icahn School of Medicine at Mount Sinai	151	Sweden

**Figure 4:**
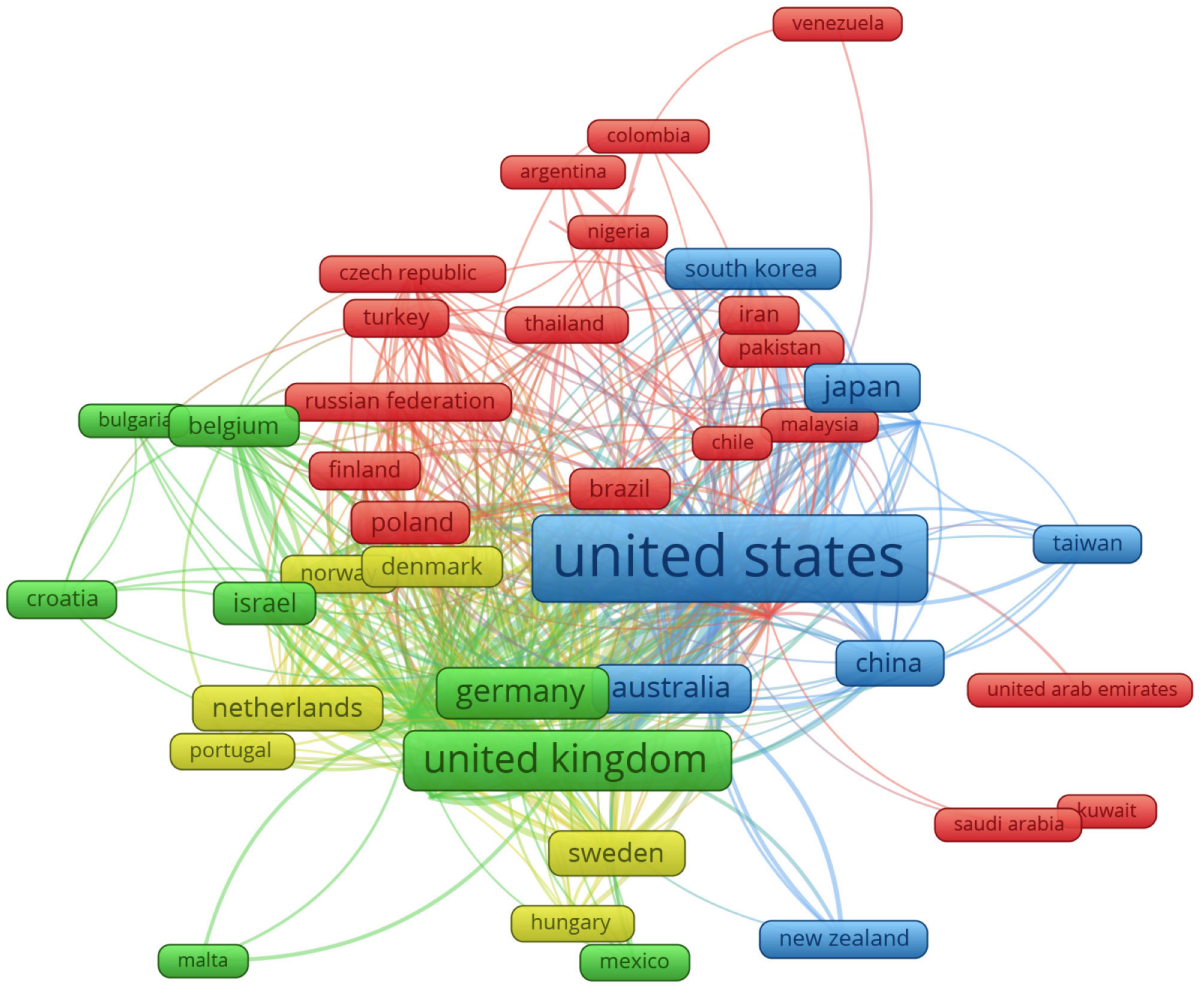
The network visualization map indicates countries’ collaboration in schizophrenia and serotonin research. Each flame represents one country. The size of the flame is proportional to the number of publications from the country, the bigger the flame and word, the higher the number of publications is. The flames with the same colour represent a cluster. A line between two flames indicates a collaboration between the two countries and at least one publication in common. Countries with at least 10 documents were used to the network visualization map.

VOSViewer was employed to produce visual representations with flames size expressing the relative number of publications and relative frequency of collaboration for 56 countries involved in joint schizophrenia–serotonin research (**Figure 4**). The bigger the flame and word, the higher the number of publications is. Additionally, both the distance between the flames (countries) and the number and thickness of the lines (links) are directly correlated with the degree of collaboration between countries. A line between two flames indicates a collaboration between the two countries represented, and thicker lines indicate stronger collaborations. A shorter distance between two countries indicates their relatedness. The VOSViewer analysis also identified four different research areas, depicted using differently coloured flames (yellow, red, blue, and green). The flames (countries) with the same colour represent a cluster.

We also examined the interactions between countries. The United States and the United Kingdom had the highest number of publications and collaborations (clusters blue and green, respectively). The main partners of the United States are the United Kingdom, Canada, and Germany. The United Kingdom is the most influential country in Europe. The main partners of the United Kingdom are the United States, Italy, and Germany. Japan is the most influential country in Asia (522 documents). The main partners of Japan are the United States, the United Kingdom, and Italy. Brazil (184 documents) is the most influential country in Latin America. In Mexico and Chile, little information is available on this topic. Brazil’s main partners are the United States, Germany, and the United Kingdom.

### 3.4 Analysis of institutes/universities

Analysing the most productive institutes and universities may provide valuable information for future institutional collaborations. **Table 2** lists the top ten institutes and universities publishing research on schizophrenia and serotonin. Five institutes are from the USA (Harvard, VA Medical Center, University of California, Yale, and NIMH), but King’s College London in the United Kingdom has the highest number of publications (304 documents). The University of Toronto in Canada (267 documents) and Harvard Medical School in the USA (262 documents) are the second and third most influential institutes investigating the schizophrenia–serotonin link.

### 3.5 Analysis of trend topics and research hotspots

**Figure 5:**
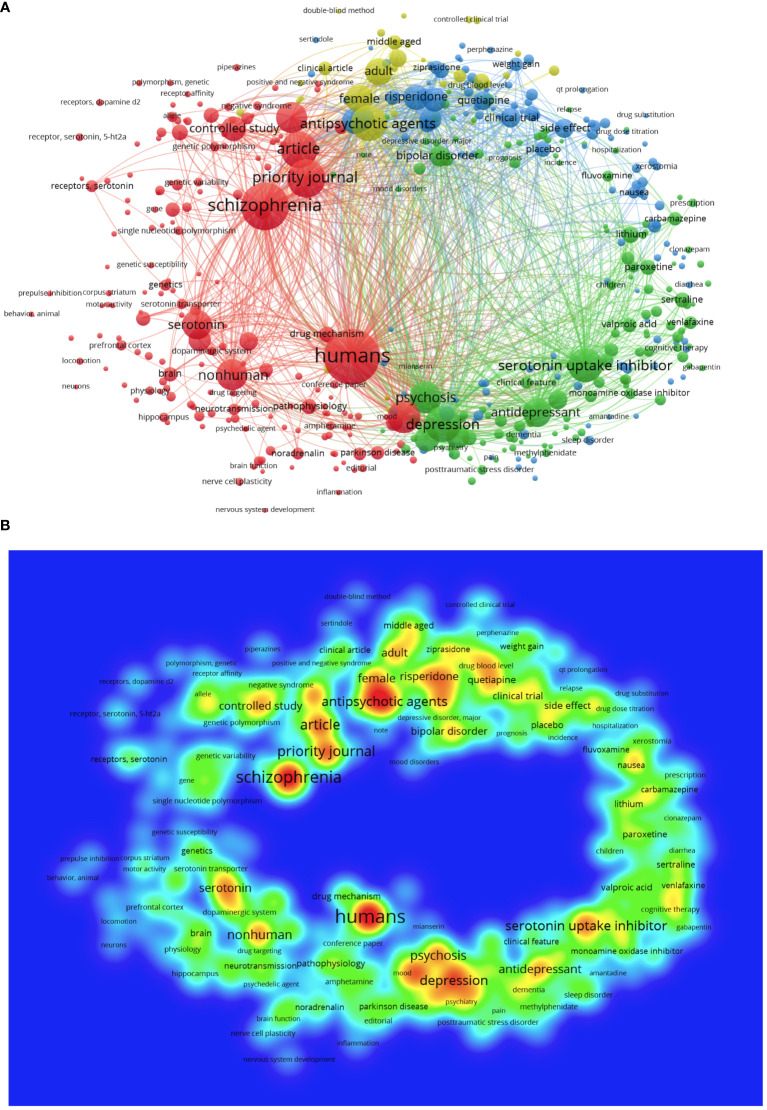
**(A)** The network visualization map indicates keyword related to schizophrenia and serotonin. Different colours represent different topic areas. **(B)** The density visualization map indicates the occurrence of keyword in the title/abstract in schizophrenia and serotonin research. The colours range from blue to green to red to indicate the frequency with which keywords appear. Red areas represent hotspots.

We analysed the occurrence of the author keywords and journal editors keywords of 12,027 documents. Among the 39,810 keywords observed, 470 appeared at least 200 times. **Figure 5A** shows a network visualisation map of keywords in the fields of schizophrenia–serotonin.

VOSViewer software can generate visualizing bibliometric maps and clusters (communities) based on the clustering algorithm and the network importance of keywords. In the map visualizations, a cycle (node) represents a keyword (item). The size of the circles indicates its importance according to the frequency of each keyword’s occurrence in published papers. Different colours indicate different clusters, and number of lines are directly correlated with the degree of relationship between keyword. **Figure 5A** shows a network visualisation map of keywords used in schizophrenia–serotonin research.

VOSviewer divided the keywords into four clusters representing different research areas. Cluster 1 (red frames) includes 181 items commonly observed in publications related to human and non-human medical research, including pathophysiology, molecular pathways, biological markers, drug targeting, and genes. Clusters 2 and 4 (green and yellow frames, respectively) include 161 items frequently observed in biomedical research on antipsychotic, antidepressant, and anxiolytic drugs, including mechanism efficacy and side effects. Cluster 3 (blue frames) includes 125 items frequently seen in research related to signs and symptoms of schizophrenia and drug classes, including serotonin reuptake inhibitor drugs, depression, comorbidities, disease course, and suicide.

**Figure 5B** shows a density visualisation map that indicates the occurrence of keywords in schizophrenia–serotonin publications. The colours, ranging from blue to red, indicate the frequency of the keywords. Keywords in the red area appear more frequently and keywords in the green area appear less frequently. The keyword ‘human’ appeared in the red area, meaning that it represents an important hotspot. Most publications related to schizophrenia–serotonin present adult human studies investigating topics including depression, antipsychotic agents, serotonin uptake inhibitors, and other mental diseases such as bipolar disorder. Conversely, ‘non-human’ studies typically focus on mouse models. Keywords such as ‘personalised medicine’, ‘alternative therapies’, ‘pharmacogenetics’, ‘epigenetic’, ‘alternative therapies’, ‘transcranial magnetic stimulation’, ‘nervous system inflammation’, ‘gut microbiome’, and ‘artificial intelligence’ received less attention. **Supplementary Table 1** lists the top ten topics and keywords related to schizophrenia–serotonin publications; *Database 2, keyword* list all the keywords that were used in our analyses.

### 3.6 Analysis of authors and author citations

**Table 3** lists the top ten authors publishing research on schizophrenia–serotonin. A total of 31,194 authors have contributed to schizophrenia–serotonin research since 1955. Of these, only 318 authors met the criteria of having published ten documents and received at least five citations. **Supplementary Figure 1** shows eight clusters (each in a different colour) representing different research areas and collaborations between authors. H. Y. Meltzer from USA, is the most influential author, with 144 documents and 11,616 citations. His main collaborators are M. Huang (China), J. L. Kennedy (Canada), and M. Horiguchi (Japan). Serretti from Italy (66 documents and 2,526 citations) and Kasper from Austria (58 documents and 1,726 citations) are the second and third most influential researchers. Meltzer is the most cited author (USA, 11,616 citations), followed by Roth (USA, 4,789 citations) and Kapur (the United Kingdom, 4,410 citations).

**Table 3:** The top ten most highly cited authors and journals publishing research associated with schizophrenia and serotonin.

Items	Rank	Author	Articles	Citations	H Index	Country
**Author**	1	Meltzer, H. Y.	144	11,616	134	United States
	2	Serretti, A.	66	2,526	83	Italy
	3	Kasper, S.	58	1,726	93	Austria
	4	Gonzalez-Maeso, J.	50	3,036	38	United States
	5	Müller, H	49	2,254	123	Germany
	6	Weizman, A	47	1,337	83	Israel
	7	Stahl, S. M.	46	1,679	71	United States
	8	Kennedy, J. L.	43	2,840	106	Canada
	9	Golimbet, V.E.	43	1,077	28	Australia
	10	Roth, B. L.	42	4,789	122	United States
Items	Rank	Journal	Articles	Citations	H Index	Country
**Journal**	1	Journal Of Clinical Psychiatry	247	15,413	228	United States
	2	Psychopharmacology	228	17,805	214	Germany
	3	Biological Psychiatry	187	15,870	356	United States
	4	Schizophrenia Research	165	9,616	199	Netherlands
	5	American Journal Of Psychiatry	161	5,373	395	United States
	6	Journal Of Clinical Psychopharmacology	155	15,522	130	United States
	7	Neuropsychopharmacology	150	16,106	249	United Kingdom
	8	Progress In Neuro Psychopharmacology And Biological Psychiatry	145	7,191	150	United States
	9	Psychiatry Research	129	3,795	171	Ireland
	10	Journal Of Psychopharmacology	120	5,642	135	United Kingdom

### 3.7 Published journals on schizophrenia-serotonin

**Table 3** lists the top ten journals publishing research on the schizophrenia–serotonin link. A total of 12,027 documents related to schizophrenia–serotonin were published in 1,971 journals. Only 239 journals met the criterion of having published at least ten documents related to schizophrenia–serotonin. The journal with the highest number of publications is the *Journal of Clinical Psychiatry* (247 documents), followed by *Psychopharmacology* (228 documents) and *Biological Psychiatry* (187 documents). *Psychopharmacology* is the most cited journal (17,805 citations), followed by *Neuropsychopharmacology* (16,106 citations) and *Biological Psychiatry* (15,870 citations). The top ten journals are classified as relating to psychiatry or pharmacology. Fifty per cent of the journals are based in the USA, including *Journal of Clinical Psychiatry, Biological Psychiatry, American Journal of Psychiatry, Journal of Clinical Psychopharmacology, and Progress in Neuro Psychopharmacology and Biological Psychiatry.* Twenty per cent of the journals are based in the United Kingdom (Neuropsychopharmacology and Journal of Psychopharmacology). Most of the journals are classified as Q1 (90%) on the SCImago Journal Rank (SJR) system.

### 3.8 Analysis of citation

We examined the most-referenced document related to the schizophrenia–serotonin association. As presented in **Table 4**, the most important document is De Hert et al. (2011), published in *World Psychiatry*, which has 1,700 citations. This article found that many physical diseases are linked to several mental disorders (including schizophrenia) and/or psychotropic treatment. De Hert et al. have the highest Impact Index Per Article at 108.8, followed by Phillips et al. (2003) with 57.8. Phillips et al. (2003) concluded that structural and functional abnormalities in neural systems necessary for emotion processing are linked with the symptoms of schizophrenia. The third most-cited article, Harrison (1999), was published in *Brain* and has accrued 1,416 citations. This critical review summarised the state of knowledge of the neuropathology of schizophrenia. The Impact Index Per Article is given by the number of times cited divided by the number of years since the article.

**Table 4:** The top ten most highly cited publications on schizophrenia and serotonin field.

Citations	Title	Impact Index	Author	Journal	Year
1,700	Physical illness in patients with severe mental disorders. I. Prevalence, impact of medications and disparities in health care	108.8	De Hert et al.	World Psychiatry	2011
1,442	Neurobiology of emotion perception II: implications for major psychiatric disorders	57.8	Phillips et al.	Biological Psychiatry	2003
1,416	The neuropathology of schizophrenia: A critical review of the data and their interpretation	42.4	Harrison	Brain	1999
1,341	Pharmacological studies of prepulse inhibition models of sensorimotor gating deficits in schizophrenia: a decade in review	50.4	Geyer et al.	Psychopharmacology	2001
1,124	Risperidone compared with new and reference antipsychotic drugs: *in vitro* and *in vivo* receptor binding	27.8	Schotte et al.	Psychopharmacology	1996
1,042	Does fast dissociation from the dopamine d(2) receptor explain the action of atypical antipsychotics?: A new hypothesis	30.3	Kapur et al.	American Journal of Psychiatry	2001
1,026	Magic shotguns versus magic bullets: selectively non-selective drugs for mood disorders and schizophrenia	41.4	Roth et al.	Nature Reviews Drug Discovery	2004
1,004	Hallucinogens	40.1	Nichols	Pharmacology and Therapeutics	2004
961	The effects of clozapine, risperidone, and olanzapine on cognitive function in schizophrenia	29.3	Meltzer et al.	Schizophrenia Bulletin	1999
906	GABAergic interneurons: implications for understanding schizophrenia and bipolar disorder	33.3	Benes et al.	Neuropsychopharmacology	2001

## 4 Discussion

Analysing the literature through manual screening (traditional literature reviews) is insufficient to manage the vast influx of scientific papers. In recent years, the number of bibliometric articles published in various journals, including the *Frontiers in Psychiatry*, has increased. Several bibliometric analyses have analysed the trends in research on the association of schizophrenia with other diseases (i.e. inflammation, oxidative stress), but none has considered serotonin and schizophrenia together. This study provides the first bibliometric analysis and visualisation related to the association between schizophrenia–serotonin. This bibliometric analysis examined 12,027 papers from the Scopus database that were originally published from the inception of the study to 2023. We presented some interesting findings that are summarised below.

Most publications related to schizophrenia–serotonin are found in journals like the *Journal of Clinical Psychiatry* and *Psychopharmacology*. As expected, most of the documents were research articles (49.38%) and review articles (34.30%).

Our study revealed that the USA is the leader in terms of the number of publications related to the schizophrenia–serotonin link. The USA is the global leader because it has high economic power, investment in medical research, as well as high expenditure on healthcare. In fact, according to the World Bank the Gross Domestic Product (GDP) of the USA is 3.4%. The USA has high prevalence rates (between 0.25% and 0.64%) of people with schizophrenia and a strong interest in the connection between schizophrenia and serotonin (26–28). Furthermore, in the US the annual cost of patients with schizophrenia was estimated to be higher compared to other diseases (i.e. cancer) (29). González-Maeso, Meltzer, Stahl, and Roth were four of the top contributors located in the USA. Five institutes from the top ten publications (Harvard, VA Medical Center, University of California, Yale, and NIMH) are located in the USA. Additionally, five of the top ten journals publishing research on schizophrenia–serotonin are based in the USA.

King’s College London in the United Kingdom is the institute that has generated the most publications in the schizophrenia–serotonin field. The Institute of Psychiatry, Psychology, and Neuroscience at King’s College London produces highly cited outputs (in the top 1% by the number of citations) in psychiatry and mental health (SciVal 2023). Likewise, this institute includes thirty of the most highly cited researchers in the fields of psychiatry and neuroscience. In the context of schizophrenia, the Department of Psychosis Studies has received over 100 research awards and contributed to creating international guidelines and policies to improve the care of people with psychosis. However, this should not overshadow the contributions of other nations. In our study, the most important document, titled *Physical illness in patients with severe mental disorders. I (2011).*, was jointly published by collaborators from different nations, including the USA, Spain, Argentina, Germany, and Netherlands. Most international collaborations on publications related to the schizophrenia–serotonin link were between institutions located in North America (USA and Canada) and Europe (the United Kingdom, Germany, and Italy).

The schizophrenia–serotonin link has received less attention in Latin America than in North America and Europe due to lower economic power and less investment in medical research. For example, research and development expenditure in Mexico represented 0.24% of the GDP, while the USA, United Kingdom, Germany, and Italy investe 3.4%, 2.9%, 3.1%, and 1.4% of their GDP, respectively. Brazil is the most productive country in Latin America and invests 1.2% of its GDP. The majority of its international collaborations were between the USA, Germany, and the United Kingdom.

### 4.1 Publications from the 1950s to the 1980s

After the first article related to schizophrenia and serotonin was published in the 1950s, research on these topics showed limited but steady growth. The amount of research increased by 12.2% between 1955 and 1969 and by 20.8% between 1970 and 1990. The growth in the 1950s was likely due to the emergence of first-generation antipsychotics in 1952 (chlorpromazine), the discovery of serotonin in the mammalian brain in 1953, and the recognition of the potential role of serotonin in nervous disorders (1954). In 1955, the spectrophotofluorimeter was introduced, the field of neuropharmacology was born, and the discovery was made that chlorpromazine possessed anti-serotonin effects. Wander Laboratories initiated the study of tricyclic compounds for antidepressant activity in 1958, and clozapine was later identified. During the 1950s and early 1960s, there was an increase in the use of the hallucinogenic drug lysergic acid diethylamide (LSD) in psychiatric research. However, during the 1960s, LSD research began to decline, serotonin-containing neurons were visualised, and Woolley suggested that schizophrenia may result from an excess of brain serotonin (30, 31).

Despite the LSD prohibition, the growth rate of articles related to schizophrenia and serotonin was 20.8% between 1970 and 1990. This increase was likely due to international collaboration among scientists. The first international study of schizophrenia, which involved researchers in New York and London, was published in 1972. Clinical and biomedical studies continued years later. Moreover, it may be that LSD research was never prohibited, but rather that changes in LSD regulation for research and development occurred (31). During the 1970s, clozapine was withdrawn from the European market. However, research on serotonin and its relation to schizophrenia’s pathogenesis continued, revealing decreased 5-HT2A receptor levels in schizophrenia, as well as increased levels of 5-HT and 5-hydroxyindoleacetic acid (5-HIAA) in subcortical brain regions of people affected. During the 1980s, the number of studies related to clozapine continued to rise.

### 4.2 Publications in the 1990s

During the 1990s, the schizophrenia–serotonin publication rate grew by 24.5%. This trend changed in the 1990s probably due to the approval of new pharmacological treatments by the US FDA, including fluoxetine (1987), clozapine (1990), risperidone (1993), and fluvoxamine (1994) (32–36), as well as the application of computed tomographic (CT), magnetic resonance imaging (MRI), and positron emission tomographic (PET) scans to the study of the structure and function of the brains of people with schizophrenia (37).

With the arrival of molecular biology methods in the 1990s, animal studies emerged as an essential methodological tool for elucidating the complex relationship between schizophrenia and serotonin (38). Research using animal models of schizophrenia revealed new treatment targets and demonstrated the potential role of serotonin in the pathophysiology of schizophrenia (39). For example, animal models based on GABA, dopamine, glutamate, serotonin, and genetic polymorphisms in dysbindin-1 (DTNBP1), disrupted-in-schizophrenia 1 (DISC1), and neuregulin 1 (NRG1) have been considered for the development of drug therapy. Novel targets like the orexin system, muscarinic and nicotinic receptors, and cannabinoid receptors have been studied through simulation of the negative and cognitive symptoms of schizophrenia. Non-pharmacological models that mimic schizophrenia’s symptoms, such as those involving social isolation, have provided a novel model for testing drug therapies. Animal models have also played a crucial role in the preclinical evaluation of the efficacy and safety of potential pharmacological treatments and the mitigation of the risk of adverse effects in human subjects (40). Therefore, the rapid growth in published papers related to schizophrenia and serotonin during the 1990s was facilitated by positive results for drugs used to treat mental illnesses (serotonin and dopamine antagonist drugs) as well as new technologies and non-invasive techniques enabling the study of the human brain in living people.

### 4.3 Publications in 2000s and 2010s

In 2000, research on the schizophrenia–serotonin link began to decrease. Between 2000 and 2008, the growth rate was reduced to 7.1%. There was an abrupt and more dramatic decrease (negative growth rate: −6.1%) from 2008 to 2019. The reason for this decline may have been the deinstitutionalisation of psychiatric patients and the change in attitudes concerning their living in society. In 1999, the US Supreme Court declared that mental illness represented a disability and was protected under the Americans with Disabilities Act (ADA). This decision established the transition of individuals with mental illness from clinical or hospital-based treatment (institutionalisation) to community-based care (deinstitutionalisation) (41). In the United States, the number of patients institutionalised in state hospitals was 165 million in 1955; by 1998, this number had fallen to 57,151 institutionalised patients (42).

### 4.4 Situation during COVID-19

During the COVID-19 pandemic, there was an increase in papers focused on the relationship between serotonin and schizophrenia submitted to health and medicine journals published by Elsevier (43). We speculate that this increase was probably due to the higher risk of severe COVID-19 infection in people with schizophrenia (44, 45). In fact, in our study, ‘COVID’ and related synonyms were common *keywords* used during 2021 (see, Database 2, keyword section). Several studies have found associations between mental illnesses, including schizophrenia, and conditions such as cardiovascular disease, diabetes mellitus, and chronic respiratory diseases like chronic obstructive pulmonary disease (COPD), as well as an increased risk for venous thromboembolism (46–49). The risk factors for severe COVID-19 infection include cardiovascular disease, metabolic disease, and chronic respiratory diseases (50). Thus, patients with schizophrenia were vulnerable to the effects of COVID-19, resulting in poorer health outcomes (i.e. higher risk of coagulopathy) (51, 52). Furthermore, recent evidence reveals that COVID‐19 is associated with several neurological disorders via an inflammatory response that triggers neuropsychiatric symptoms, including schizophrenia, via immunological processes (53). In fact, one long‐term effect of COVID‐19 could be an increased risk of schizophrenia (53). But further research is needed to establish this relationship.

The use of SSRIs increased during the COVID-19 pandemic. This was due to their use in the early stages of COVID-19 to reduce complications such as intubation and death (54, 55). Various mechanisms could explain the protective effects of antidepressant drugs against COVID-19 infection. The use of SSRIs could reduce complications in COVID-19 infection by inhibiting serotonin transporter, decreasing mast cell degranulation, interfering with the trafficking of the virus, inhibiting acid sphingomyelinase, increasing levels of melatonin, acting as an agonist for the sigma-1 receptor, and providing anti-inflammatory effects (55, 56).

We anticipate that the scientific community will continue to be interested in topics related to schizophrenia–serotonin and novel hypotheses on the origin of schizophrenia. We speculate that the growth rate for schizophrenia–serotonin papers is projected to stay at 3.1% in 2033 and 2.6% in 2040.

### 4.5 Areas of interest and hotspots

The most frequently used keywords in published papers were ‘humans’, ‘schizophrenia’, and ‘antipsychotic agents’. Our study revealed that ‘adult’ was a more frequent keyword compared to ‘adolescent’ and ‘children’. Schizophrenia’s symptoms usually manifest in late adolescence or adulthood and less frequently in children. First, the brain continues developing until the age of 20, and diagnosing schizophrenia in children and adolescents is more difficult because the symptoms are less evident in children than in adults (57). Furthermore, the risk of developing schizophrenia is elevated during infancy and adolescence due to the interaction between genetic and environmental factors, such as childhood trauma, disruptions in circadian rhythms, or social isolation (58). The most commonly diagnosed mental illnesses in infants and adolescents are anxiety, depression, eating disorders, attention deficit hyperactivity disorder, and conduct disorders (59). It is interesting to note that the ‘no human model’ keywords ranked in eighth place. Notwithstanding this result, we speculate future research will probably continue to include ‘no human model’ (rodent model) to elucidate the molecular mechanisms and pathophysiology of schizophrenia.

Although there are many ‘antipsychotic agents’ currently available, one in three patients do not respond to classical treatment (pharmacologic and psychological interventions) (60). Thus, the development of new drugs for schizophrenia represents a significant challenge. There are new drugs with diverse mechanisms of action (i.e. lumateperone) and specific drugs (i.e. pimavanserin indicated for psychosis caused by Parkinson’s disease) on the market. Additionally, a variety of novel drugs are currently in the clinical trial investigation for the treatment of schizophrenia, including Brilaroxazine, Xanomeline/Trospium, Emraclidine, Ulotaront, Sodium Benzoate, Luvadaxistat, and Iclepertin (61).

Future diagnostic criteria should include results from studies on genetic risk, neuropsychological, and cranial neuroimaging. We suggest that researchers shift their focus to emerging themes. Advancements in personalised medicine including pharmacogenetics, epigenetics, pharmacotherapy, psychotherapy techniques, alternative therapies such as transcranial magnetic stimulation, as well as the study of alternative pathogenesis of schizophrenia (inflammation, gut microbiome), the optimization of current treatment (therapeutic drug monitoring, pharmacogenetic testing, and 3D printing drugs) and the integration of artificial intelligence (AI), will probably improve medical interventions and the diagnosis of individuals with schizophrenia. For instance, the use of brain scans and AI during diagnostic testing could distinguish between patients who require immediate attention and those who may safely wait to receive treatment (62). Moreover, in the ‘omic’ era new terms such as ‘serotoninomic’ have emerged. Serotoninomic concept was coined in 2015, which encompasses all studies carried out exclusively on serotonin and its system, including the experimental techniques and laboratory tools to contribute to finding precise answers regarding basic, clinical, and translational research related to serotonin, just as the emerging medical and ‘omics’ sciences have done (63, 64).

**Figure 6:**
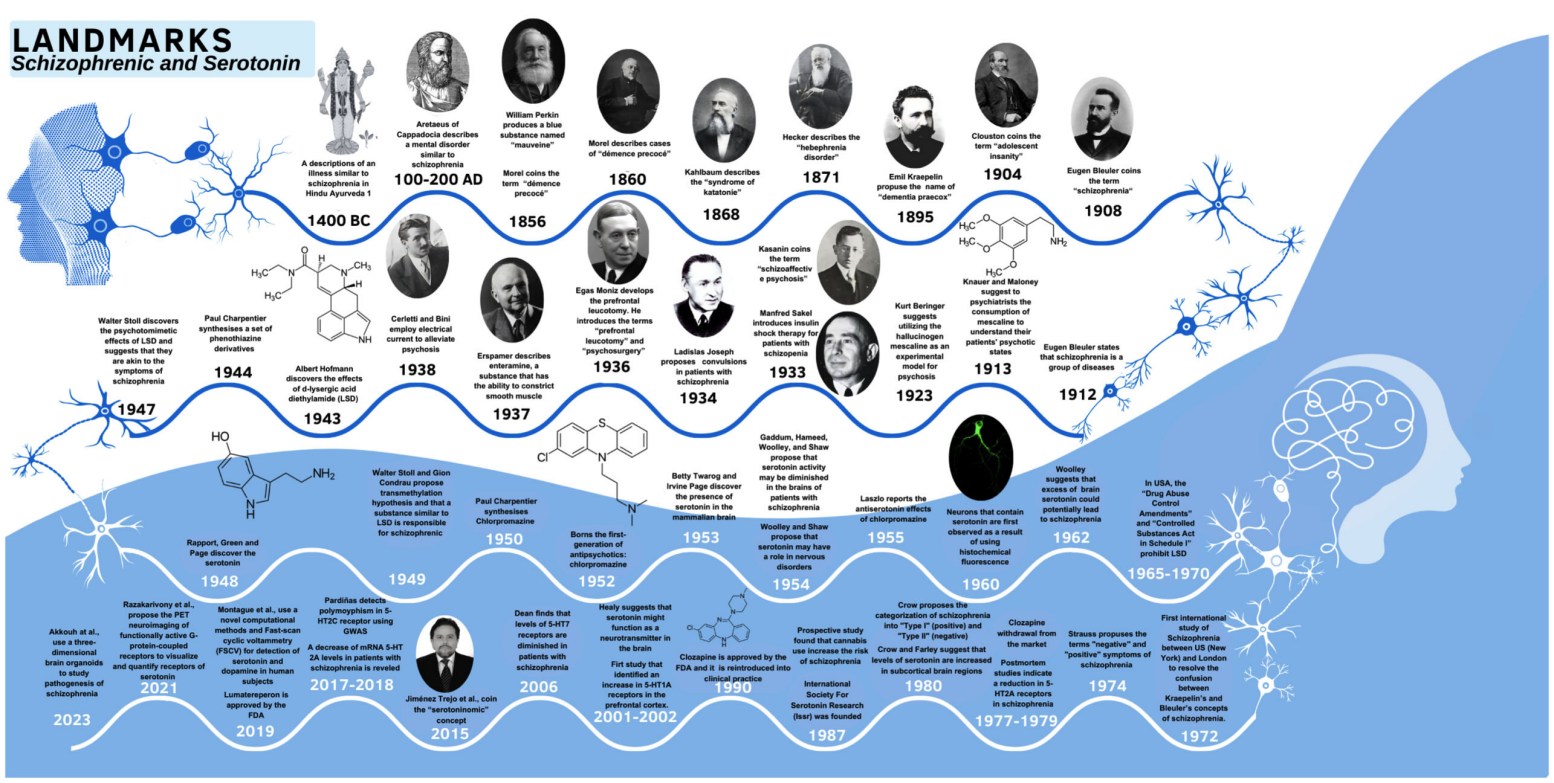
Timeline showing key dates and landmark discoveries in serotonin-schizophrenia field.

The understanding of the relationship between schizophrenia and serotonin and the field has changed over time. **Figure 6** shows the history in this field, beginning with the ancient Hindu scriptures and the work of Aretaeus of Cappadocia in the Greek period. We appreciate the current state of schizophrenia and serotonin achievement, the recognition of terms such as schizophrenia (65) and serotoninomic (63, 64), the discovery of serotonin and hallucinogens (mescaline and d-lysergic acid diethylamide) (66–68), the introduction of treatments for patients with schizophrenia, the development of first-generation antipsychotics (69), and the understanding of the serotonin system’s involvement in individuals with schizophrenia.

Even though we obtained some fascinating results using bibliometric analysis, our study has some limitations. One big limitation is that non-English language articles were excluded. Second, the data used in our bibliometric analysis were downloaded exclusively from Scopus, meaning that articles published in other database or unindexed journals were excluded. Third, we used the terms ‘schizophrenia’ and ‘serotonin’ including in the author keywords and journal editors keywords as our search method (keyword search). Thus, articles containing ‘schizophrenia’ and ‘serotonin’ in the abstract, title or text but not in the Keywords would have been excluded from our study. Thus, this search method often discards a large portion of potentially relevant data. Articles with different author keywords or journal editors keywords (such as dopamine) were also omitted.

Another limitation of bibliometric analysis is that it does not assess the content of individual articles in depth due to the large size of the database. Systematic reviews and meta-analyses are considered the highest level of evidence in medicine. Meta-analyses are considered original research articles by most journals, while systematic reviews are more specific and focused compared to the standard literature review, which tends to be more general. We were unable to determine information regarding the number of meta-analyses or systematic reviews included in our study. Nevertheless, we observed that the keywords ‘systematic review’ and ‘meta-analyses’ ranked 182nd and 170th, respectively. Moreover, ‘systematic review’ and ‘meta-analyses’ were keywords used mainly during 2014 and 2011, respectively (see, Database 2, keyword).

## 5 Conclusions

From ancient times to the present day, there has been a gradual progression of knowledge regarding schizophrenia and serotonin. In the last twenty years, there has been an increase in academic papers in this field. The COVID-19 pandemic has created an extraordinary opportunity—not only to advance in this and other psychiatric illnesses but also to be able to determine how to mitigate them through collaboration with the scientific and medical community. Future investigations should underscore the importance of more systematic reviews and meta-analyses, multidisciplinarity, and collaboration between different authors and universities in understanding the physiopathology of schizophrenia and the function of serotonin. We believe that the development of novel medications and treatments for schizophrenia will be increased in the future, as well as research into genetic risks, psychological factors, and cranial neuroimaging components. Future schizophrenia and serotonin research is likely to focus on personalised medicine, alternative therapies, novel pathogenesis of schizophrenia, and the use of emerging technologies such as artificial intelligence.
