# Corrigendum: Global research in schizophrenia and serotonin: a bibliometric analysis

**DOI:** 10.3389/fpsyt.2024.1496529

**Published:** 2024-09-27

**Authors:** Gustavo Canul-Medina, Gael López-Pech, Francisco Jiménez-Trejo

**Affiliations:** ^1^ School of Medicine, Educational Center Rodriguez Tamayo, Ticul, Yucatan, Mexico; ^2^ Cellular and Tissue Morphology Laboratory, National Institute of Pediatrics, Mexico City, Mexico

**Keywords:** schizophrenia, serotonin, bibliometric analysi, scopus, VOSviewer

In the published article, there was an error in the **Funding** statement. The grant number was reported incorrectly as “034/2015”. The correct statement appears below:

“The author(s) declare financial support was received for the research, authorship, and/or publication of this article. The project was supported by grant 046/2019, obtain from Federal Resources E-022 of the Instituto Nacional de Pediatría, Laboratorio de Morfología Celular y Tisular, Ciudad de México, México.”

The authors apologize for this error and state that this does not change the scientific conclusions of the article in any way. The original article has been updated.

