# Supplementary material for: Global research in schizophrenia and serotonin: a bibliometric analysis
Source: Front Psychiatry. 2024 Aug 2;15:1436906. doi: 10.3389/fpsyt.2024.1436906 (PMC11329940; doi:10.3389/fpsyt.2024.1436906)
Supplement: Supplementary file 1 [file Table1.docx]

Supplementary Material

**Global Research in Schizophrenia and Serotonin: A Bibliometric Analysis**

Gustavo Canul-Medina*, Gael Lopez-Pech, Francisco Jiménez-Trejo

*** Correspondence:** Francisco Jiménez-Trejo. e-mail: trejofjj@gmail.com

# Supplementary Figures and Tables

## Supplementary Tables: The top ten topics/keywords related to schizophrenia and serotonin publications.

| **Rank** | **Keyword** | **Frequency** | **Total link strength** |  |
| --- | --- | --- | --- | --- |
| 1 | Humans | 10,727 | 2,647,75 |  |
| 2 | Schizophrenia | 8,268 | 1,892,36 |  |
| 3 | Antipsychotic  agents | 5,243 | 1,466,39 |  |
| 4 | Depression | 5,170 | 1,434,54 |  |
| 5 | Serotonin uptake  inhibitor | 4,757 | 1,373,45 |  |
| 6 | Psychosis | 4,205 | 1,172,38 |  |
| 7 | Male | 4,097 | 90,960 |  |
| 8 | Nonhuman | 4,044 | 95,912 |  |
| 9 | Serotonin | 3,800 | 75,172 |  |
| 10 | Antidepressant | 3,589 | 1,111,34 |  |

## Supplementary Figures: Authors of schizophrenia and serotonin publications.


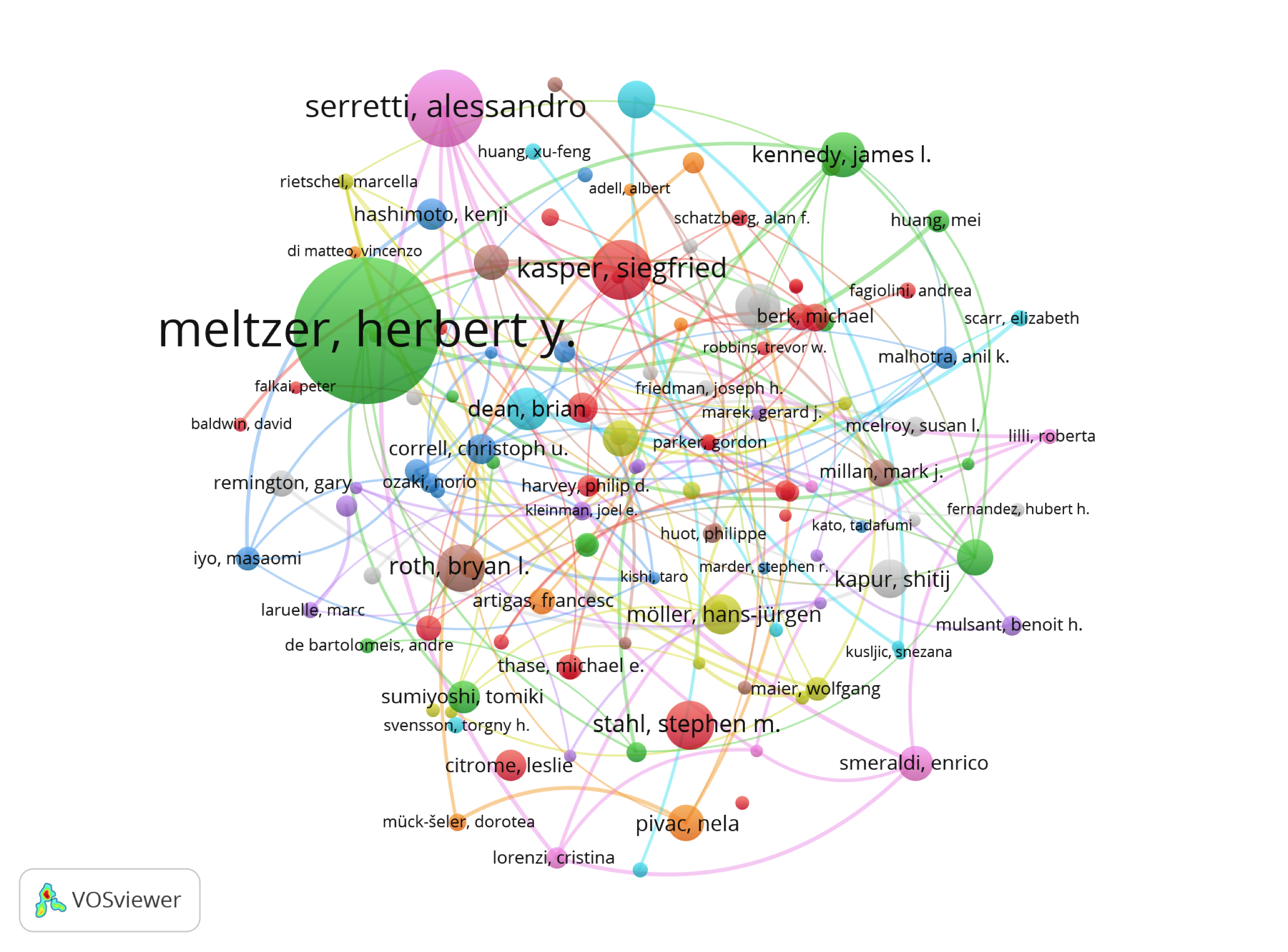


## Supplementary Table: Threshold values and visualization methodologies for each type of analysis in VOSviewer.

| **Type of Analysis** | **Unit of analysis** | **Analysis** | **Visualization** |
| --- | --- | --- | --- |
| Co-autorship: country/region | - Minimum 10 documents by country - Minimum 3 citations by country | - Method: association - Layout attraction: 5 - Layout repulsion: 1 - Clusterig resolution: 1 - Minimun cluster size: 1 | - Scale - Weight: document - Max length:30 - Size variation: 0.5 - Min strength: 3 - Max line: 1300 |
| Co-ocurrence: all keyword | - Minimum 200 occurrence of a keyword - keywords eliminated and thesaurus check annexes 1 | - Method: association - Layout attraction: 5 - Layout repulsion: 2 - Clusterig resolution: 1 - Minimun cluster size: 10 | - Weight: occurrence - Scale:0.81. Ocurrence - Max length:26 - Size variation: 0.60 - Min strength: 1 - Max line: 1000 - Color density: rainbow - Density kernel width: 0.5 - Item Density |
| Co-autorship: authors | - Minimum 10 documents by author - Minimum 5 citations by author | - Method: linlong modularity - Layout attraction: 4 - Layout repulsion: -2 - Clusterig resolution: 1   Minimun cluster size: 2 | - Weight: document - Max length:26 - Size variation label: 0.83 - Max line: 1000 - Min strength: 1 - Size variation line: 0.84 - Visualization Scale:1.27 |
| Bibliographic coupling analysis: documents | - Minimum 50 citation by documents | - Method: linlong modularity - Layout attraction: 2 - Layout repulsion: -7 - Clusterig resolution: 1   Minimun cluster size: 5 | - Weight: citation - Max length:26 - Size variation label: 0.94 - Max line: 1000 - Min strength: 2 - Size variation line: 0.88 - Visualization Scale:1.15 |
| Citation: source | - Minimum 10 documents by source - Minimum 1 citations by author | - Method: linlong modularity - Layout attraction: --5 - Layout repulsion: -6 - Clusterig resolution: 1   Minimun cluster size: 2 | - Visualization Scale:1.27 - Size variation line: 0.84 - Weight: document - Max length:26 - Size variation label: 0.83 - Max line: 1000 - Min strength: 1 |
| Citation: source | - Minimum 10 documents by source - Minimum 1 citations by author |  |  |

## Supplementary Table: Reference of Figure 6 Timeline

| 1400 BC | (1) |
| --- | --- |
| 100-200 AD | (2) |
| 1856 | (3,4) |
| 1960 | (5) |
| 1868 | (6) |
| 1871 | (7) |
| 1895 | (8) |
| 1904 | (9) |
| 1908 | (10) |
| 1912 | (11) |
| 1923 | (12) |
| 1933 | (13) |
| 1933 | (14) |
| 1934 | (14) |
| 1936 | (15) |
| 1938 | (14) |
| 1943 | (16) |
| 1944 | (17) |
| 1947 | (16) |
| 1948 | (18) |
| 1949 | (19) |
| 1949 | (20) |
| 1950 | (21) |
| 1950 | (22,23) |
| 1952 | (24) |
| 1953 | (25) |
| 1954 | (26) |
| 1954 | (27) |
| 1954 | (26,28) |
| 1955 | (29) |
| 1956 | (30) |
| 1958 | (31) |
| 1960 | (32) |
| 1962 | (33) |
| 1972 | (34) |
| 1974 | (35) |
| 1977-1979 | (36,37) |
| 1979-1980 | (38)(39) |
| 1980 | (40) |
| 1987 | (41) |
| 1988 | (42,43) |
| 1990 | (31) |
| 2001-2002 | (44,45) |
| 2002 | (46) |
| 2006 | (47) |
| 2015 | (48,49) |
| 2017 | (50) |
| 2018 | (51) |
| 2023 | (52) |
| 2019 | (53) |
| 2024 | (54) |

**References**

1. Lakshmikuttyamma A, Hajjar E, Henley C, Lungen JM. Antipsychotic agents. *Side Effects of Drugs Annual*. (2022) 44:7–16. doi: 10.1016/BS.SEDA.2022.09.016

2. Kauffman P. Did Schizophrenia Exist in Ancient Greece and Rome?: Schizophrenia and Epigenetics. *The International Journal of Health, Wellness, and Society*. (2017) 7. doi: 10.18848/2156-8960/cgp/v07i04/9-23

3. Wender PH. Dementia praecox: the development of the concept. *Am J Psychiatry*. (1963) 119. doi: 10.1176/ajp.119.12.1143

4. Cunningham Owens D, Johnstone EC. The development of antipsychotic drugs. *Brain Neurosci Adv*. (2018) 2. doi: 10.1177/2398212818817498

5. Morel BA. Traité des maladies mentales: par B.A. Morel [Internet]. V. Masson; 1860.

6. Barnes MP, Saunders M, Walls TJ, Saunders I, Kirk CA. The syndrome of karl ludwig kahlbaum. *J Neurol Neurosurg Psychiatry*. (1986) 49. doi: 10.1136/jnnp.49.9.991

7. Kraam A. Classic Text No. 77: “Hebephrenia. A contribution to clinical psychiatry” by Dr. Ewald Hecker in Görlitz (1871). Vol. 20, History of Psychiatry. 2009. doi: 10.1177/0957154X08099416

8. Adityanjee, Aderibigbe YA, Theodoridis D, Vieweg WVR. Dementia praecox to schizophrenia: The first 100 years. Vol. 53, Psychiatry and Clinical Neurosciences. 1999. doi: 10.1046/j.1440-1819.1999.00584.x

9. Clouston TS. Clinical lectures on mental diseases. HC Lea’s Son; 1884.

10. Ashok AH, Baugh J, Yeragani VK. Paul Eugen Bleuler and the origin of the term schizophrenia (SCHIZOPRENIEGRUPPE). *Indian Journal of Psychiatry*. (2012) 54. doi: 10.4103/0019-5545.94660

11. Bleuler E, Brill AA (Abraham A. Textbook of psychiatry / by Eugen Bleuler ; [authorized English ed. by A.A. Brill]. Special ed. New York: Classics of Psychiatry & Behavioral Sciences Library; 1988. (The Classics of psychiatry & behavioral sciences library).

12. Beringer K. Experimentelle psychosen durch mescalin. Vortrag, gehalten auf der südwestdeutschen psychiaterversammlung in Erlangen 1922. *Zeitschrift für die gesamte Neurologie und Psychiatrie*. (1923) 84. doi: 10.1007/BF02896052

13. The acute schizoaffective psychoses. 1933 [classical article]. *American Journal of Psychiatry*. (1994) 151. doi: 10.1176/ajp.151.6.144

14. Eddington S, Pal R, Cheng T, Zorumski CF, Cristancho P. Electroconvulsive Therapy. *Reference Module in Neuroscience and Biobehavioral Psychology* [Internet]. (2023) [cited 2024 Jun 26]. Available from: https://linkinghub.elsevier.com/retrieve/pii/B9780323957021000178doi: 10.1016/B978-0-323-95702-1.00017-8

15. Solomon HC. Prefrontal leukotomy, an evaluation. *Journal of the American Medical Association*. (1949) 140. doi: 10.1001/jama.1949.02900480009003

16. Stoll WA. Lysergsaure-diathylamid, ein Phantastikum aus der Mutterkorngruppe. Royal Society of Medicine, Microfilm unit.; 1947.

17. CHARPENTIER P. On the constitution of a dimethylamino-propyl-N-phehiazine. *Comptes rendus hebdomadaires des seances de l’Academie des sciences*. (1947) 225:306–8.

18. Rapport MM, Green AA, Page IH. Serum vasoconstrictor, serotonin; isolation and characterization. *The Journal of biological chemistry*. (1948) 176.

19. RAPPORT MM. Serum vasoconstrictor (serotonin) the presence of creatinine in the complex; a proposed structure of the vasoconstrictor principle. *The Journal of biological chemistry*. (1949) 180.

20. Nichols DE, Walter H. The History of Psychedelics in Psychiatry. Vol. 54, Pharmacopsychiatry. 2021. doi: 10.1055/a-1310-3990

21. Jablensky A. The diagnostic concept of schizophrenia: its history, evolution, and future prospects. *Dialogues in Clinical Neuroscience*. (2010) 12. doi: 10.31887/dcns.2010.12.3/ajablensky

22. Bennett MR. Monoaminergic synapses and schizophrenia: 45 Years of neuroleptics. Vol. 12, Journal of Psychopharmacology. 1998. doi: 10.1177/026988119801200310

23. Deniker P. Introduction of neuroleptic chemotherapy into psychiatry. *Discoveries in biological psychiatry*. (1970):155–64.

24. Ban TA. Fifty years chlorpromazine: A historical perspective. Vol. 3, Neuropsychiatric Disease and Treatment. 2007.

25. TWAROG BM, PAGE IH. Serotonin content of some mammalian tissues and urine and a method for its determination. *The American journal of physiology*. (1953) 175. doi: 10.1152/ajplegacy.1953.175.1.157

26. Woolley DW, Shaw E. A biochemical and pharmacological suggestion about certain mental disorders. *Proceedings of the National Academy of Sciences*. (1954) 40. doi: 10.1073/pnas.40.4.228

27. Some Neurophysiological Aspects of Serotonin. *British Medical Journal*. (1954) 2. doi: 10.1136/bmj.2.4880.122

28. GADDUM JH, HAMEED KA. Drugs which antagonize 5-hydroxytryptamine. *British journal of pharmacology and chemotherapy*. (1954) 9. doi: 10.1111/j.1476-5381.1954.tb00848.x

29. Gyermek L. Chlorpromazine: A serotonin antagonist? *The Lancet*. (1955) 266:724.

30. GYERMEK L, LAZAR I, CSAK AZ. The antiserotonin action of chlorpromazine and some other phenothiazine derivatives. *Archives internationales de pharmacodynamie et de thérapie*. (1956) 107.

31. Hippius H. A historical perspective of clozapine. *J Clin Psychiatry*. (1999) 60:22–3.

32. Dahlström A, Fuxe K. Localization of monoamines in the lower brain stem. *Experientia*. (1964) 20. doi: 10.1007/BF02147990

33. Quednow BB, Geyer MA, Halberstadt AL. Serotonin and schizophrenia. *Handbook of Behavioral Neuroscience*. (2020) 31:711–43. doi: 10.1016/B978-0-444-64125-0.00039-6

34. Cooper JE, al et. Psychiatric diagnosis in New York and London: A comparative study of mental hospital admissions. Psychiatric diagnosis in New York and London: A comparative study of mental hospital admissions. 1972.

35. Kirkpatrick B, Fernandez-Egea E. Assessment and the concept of negative symptoms. *Spanish Journal of Psychiatry and Mental Health*. (2024). doi: 10.1016/j.sjpmh.2023.09.006

36. Bennett JP, Enna SJ, Bylund DB, Gillin JC, Wyatt RJ, Snyder SH. Neurotransmitter Receptors in Frontal Cortex of Schizophrenics. *Archives of General Psychiatry*. (1979) 36. doi: 10.1001/archpsyc.1979.01780090013001

37. Amsler HA, Teerenhovi L, Barth E, Harjula K, Vuopio P. Agranulocytosis in patients treated with clozapine: A STUDY OF THE FINNISH EPIDEMIC. *Acta Psychiatrica Scandinavica*. (1977) 56. doi: 10.1111/j.1600-0447.1977.tb00224.x

38. Farley IJ, Shannak KS, Hornykiewicz O. Brain monoamine changes in chronic paranoid schizophrenia and their possible relation to increased dopamine receptor sensitivity. *Advances in biochemical psychopharmacology*. (1980) 21.

39. Crow TJ, Baker HF, Cross AJ, Joseph MH, Lofthouse R, Longden A, et al. Monoamine mechanisms in chronic schizophrenia: Post-mortem neurochemical findings. *British Journal of Psychiatry*. (1979) 134. doi: 10.1192/bjp.134.3.249

40. Crow TJ. Molecular pathology of schizophrenia: More than one disease process? *British Medical Journal*. (1980) 280. doi: 10.1136/bmj.280.6207.66

41. Andréasson S, Engström A, Allebeck P, Rydberg U. CANNABIS AND SCHIZOPHRENIA A Longitudinal Study of Swedish Conscripts. *The Lancet*. (1987) 330. doi: 10.1016/S0140-6736(87)92620-1

42. Kane J, Honigfeld G, Singer J, Meltzer H. Clozapine for the Treatment-Resistant Schizophrenic: A Double-blind Comparison With Chlorpromazine. *Archives of General Psychiatry*. (1988) 45. doi: 10.1001/archpsyc.1988.01800330013001

43. Claghorn J, Honigfeld G, Abuzzahab FS, Wang R, Steinbook R, Tuason V, et al. The risks and benefits of clozapine versus chlorpromazine. *Journal of Clinical Psychopharmacology*. (1987) 7. doi: 10.1097/00004714-198712000-00002

44. Weinberger DR, Laruelle M. Neurochemical and neuropharmacological imaging in schizophrenia. In: Neuropsychopharmacology - 5th Generation of Progress. 2001.

45. Bantick RA, Deakin JFW, Grasby P. The 5-HT1A receptor in schizophrenia: A promising target for novel atypical neuroleptics? Vol. 15, Journal of Psychopharmacology. 2001. doi: 10.1177/026988110101500108

46. Gaver DL. The Creation of Psychopharmacology. *The Journal of Clinical Psychiatry*. (2005) 66. doi: 10.4088/jcp.v66n1020a

47. Dean B, Pavey G, Thomas D, Scarr E. Cortical serotonin7, 1D and 1F receptors: Effects of schizophrenia, suicide and antipsychotic drug treatment. *Schizophrenia Research*. (2006) 88. doi: 10.1016/j.schres.2006.07.003

48. Jiménez-Trejo F, Tapia-Rodríguez M, Arriaga-Canon C, Herrera LA, Contreras-Espinosa L, Jiménez-García KL. Expanding the concept of serotoninomics: perspectives for serotonin studies in the 20’s of the 21st century. *Front Neurosci*. (2023) 17:1200370. doi: 10.3389/fnins.2023.1200370

49. Jiménez-Trejo F, Rodríguez MT. Exploring the Frontiers of Serotoninomics in Male Reproduction: The Future Ahead. *Single Cell Biology*. (2015) 04. doi: 10.4172/2168-9431.1000115

50. Cheah SY, Lawford BR, Young RM, Morris CP, Voisey J. mRNA expression and DNA methylation analysis of serotonin receptor 2A (HTR2A) in the human schizophrenic brain. *Genes*. (2017) 8. doi: 10.3390/genes8010014

51. Pardiñas AF, Holmans P, Pocklington AJ, Escott-Price V, Ripke S, Carrera N, et al. Common schizophrenia alleles are enriched in mutation-intolerant genes and in regions under strong background selection. *Nature Genetics*. (2018) 50. doi: 10.1038/s41588-018-0059-2

52. Razakarivony O, Newman-Tancredi A, Zimmer L. Towards in vivo imaging of functionally active 5-HT1A receptors in schizophrenia: concepts and challenges. *Transl Psychiatry*. (2021) 11:22. doi: 10.1038/s41398-020-01119-3

53. Moran RJ, Kishida KT, Lohrenz T, Saez I, Laxton AW, Witcher MR, et al. The Protective Action Encoding of Serotonin Transients in the Human Brain. *Neuropsychopharmacology*. (2018) 43. doi: 10.1038/npp.2017.304

54. Kossatz E, Diez-Alarcia R, Gaitonde SA, Ramon-Duaso C, Stepniewski TM, Aranda-Garcia D, et al. G protein-specific mechanisms in the serotonin 5-HT(2A) receptor regulate psychosis-related effects and memory deficits. *Nature communications*. (2024) 15:4307. doi: 10.1038/s41467-024-48196-2
